# Words for clinical use in speech-language pathology in Brazilian portuguese-speaking children: a focus on familiarity and representativeness from expert judges

**DOI:** 10.1590/2317-1782/e20250307en

**Published:** 2026-07-31

**Authors:** Yanela Paola Jaimes Parada, Vitor Cantele Malavolta, Denis Altieri de Oliveira Moraes, Simone Nicolini de Simoni, Márcia Keske-Soares

**Affiliations:** 1 Programa de Pós-graduação em Distúrbios da Comunicação Humana, Universidade Federal de Santa Maria – UFSM - Santa Maria (RS), Brasil.

**Keywords:** Language Development, Child Language, Vocabulary, Language Tests, Validation Study

## Abstract

**Purpose:**

To provide a word bank based on familiarity and representativeness for the 18- to 36-month age group, considering the Brazilian cultural context.

**Methods:**

This was a cross-sectional study with a quantitative design. The questionnaire, sent to expert judges, was based on the list of words and sentences from the MacArthur-Bates Communicative Development Inventories (MB-CDIs). Three groups of experts participated: experienced speech therapists, speech therapists, and mothers with no training in the area. The questionnaire included 21 questions on familiarity and 13 on representation, which were answered using a Likert scale on familiarity (familiar or unfamiliar) and representativeness (representation by pictures or not). The responses were analyzed using descriptive and inferential statistics and visually represented through word clouds.

**Results:**

According to the criteria, only those that met the mean values were selected, leaving a total of 146 words. Based on the list from the MB-CDIs inventory adapted to Portuguese, the words were organized by category to generate the word bank, called the Word Bank for Childhood Language Development (BPDLI): A Validated Instrument for Brazilian Portuguese.

**Conclusion:**

This work provides a validated tool that can be used in clinical language assessment and intervention at early ages, allowing for greater sensitivity to the child's cultural and family context. This word bank also has therapeutic potential for approaches to phonology, narrative language, and augmentative and alternative communication.

## INTRODUCTION

The neurobiological development of human beings influences verbal language processes. From the sensory standpoint, mainly through auditory stimulation, from birth onward individuals are exposed to sound stimuli that constitute the starting point for the acquisition of any child’s verbal repertoire^(1)^.

Moreover, during the first years of life, this development takes place through the experiences the child has within his or her own context; interaction allows greater life experiences to be represented in increased lexical growth^(2)^.

This connection has been described in the scientific literature through networks that carry meanings associated with new learning. Although many children achieve age-appropriate language development, for others it represents a challenge^(3)^.

While it is true that, even before birth, children possess the essential prerequisites to develop a communicative system, during childhood the greatest uptake of phonological stimuli is achieved, and these later contribute sequentially to many functional processes involved in speech development^(4)^.

Learning new words is directly proportional to the experiences lived within the family and ends up becoming the starting point for a broad vocabulary rich in productions. These experiences are the first source of meaning, which over time will be exposed to new contexts and therefore to new challenges, according to the environment defined in this study as a symbolic transactional process^(3,5)^.

At present, there has been growing interest in creating tools in all areas of speech-language pathology to support professionals who focus on the typical and atypical aspects that influence language. It is also essential to involve families, since they are the first to detect whether there is any anomaly.

In the field of audiology, a study was conducted in Brazil on the translation and cultural validation of the Early Listening Function (ELF) instrument^(6)^. It was designed to be applied at home as an initial step in hearing assessment. This contribution makes it possible to incorporate the family as a receptive agent in the early detection of auditory behaviors and thus encourages parents to remain attentive to situations in the home.

On the other hand, it is evident that within speech-language evaluation in the area of speech, the importance of expanding studies on the phonological component continues to grow.

It is essential for a phonological repertoire to be broad enough to include all speech sounds, as shown by a study based on the search for the relationship between words that do and do not form part of the Child Phonological Assessment (Avaliação Fonológica da Criança - AFC); this work highlights the phonological evaluation of children while taking gender and age into account^(4)^.

Another important contribution to the early identification of children in Brazil with possible speech difficulties came from an international instrument, Profiles of Early Expressive Phonological Skills – Brazilian Portuguese (PEEPS-BP), which was translated and cross-culturally validated, thereby contributing to the selection of instruments appropriate for the Brazilian population^(7)^.

For the construction of instruments to assess children’s vocabulary, word selection takes into account familiarity and cultural relevance. For example, in the creation of an assessment tool for children aged 3 to 7 years, words common across different regions and cultural contexts of Brazil were selected, ensuring that they were representative and easily recognizable to children^(8)^.

Taking into account the diverse and broad variables, as demonstrated by some of the studies conducted in the phonological field in Brazil^(4,6-8)^, interest has increased in improving assessment tools, especially at early ages and taking into account the vocabulary and phonology specific to the population to which they are applied. These studies establish the importance of properly selecting words, since this determines the effectiveness of what is intended, generating benefits based on semantic maps, among other variables, so that word creation is not superficial^(9,10)^.

In addition, studies have shown that children’s performance on speech perception tasks improves significantly when lists of words familiar to them are used, compared with standardized lists^(4)^. This highlights the importance of considering the familiarity and representativeness of words in speech-language assessments.

The objective of this study is to provide a word bank based on the familiarity and representativeness of words for the 18- to 36-month age range.

## METHODS

This study is characterized as a cross-sectional investigation with a quantitative design, involving the participation of expert and non-expert judges for the development of a Word Bank for Child Language Development (BPDLI): A Validated Instrument for Brazilian Portuguese. The BPDLI was based on the familiarity and frequency of words produced by children aged 18 to 36 months, according to caregiver reports. The study is observational in nature and was written in accordance with the STROBE Statement.

This research met the required standards, as it was approved by the Ethics Committee (CEP) of a higher education institution under approval number 3.505.454. In addition, participants signed informed consent forms authorizing their participation. The research was conducted in accordance with Resolution 466/12, taking into account the requirements for data protection.

### Participants

The sample consisted of 3 types of expert and non-expert judges, for a total of 21 judges. Among the selection criteria for the group of judges, it was considered that they be located nationwide and reside in any of the states of Brazil, and that their professional practice be in the field of speech and language, as well as, of course, their experience with children in the 18- to 36-month age range. They were organized as follows:

- Group 1 – Expert judges: Specialists in the field of Speech-Language Pathology. They held doctoral degrees and had a minimum of 5 years of professional experience.
- Group 2 – Mothers who are speech-language pathologists: This group consisted of professionals in Speech-Language Pathology who also had the experience of being mothers of children between 18 and 36 months of age.
- Group 3 – Mothers who are not speech-language pathologists: This group consisted of mothers of children between 18 and 36 months of age who had neither knowledge nor professional background in the field of Speech-Language Pathology.

### Materials

The questionnaire sent to the specialist judges was based on the word list from the Communicative Development Inventory (CDI’s), words and sentences. Authorized version of the MacArthur-Bates Communicative Inventory, Brazilian version – PART I – Use of words – Vocabulary list^(11)^ It was structured in question format so that each judge could select according to his or her own criteria. This electronic questionnaire consisted of a total of 480 words.

### Procedures

To collect the data, the three groups of judges were contacted and sent, by email, forms created in Google Docs, inviting them to act as expert judges and contribute to the selection of words based on familiarity and representativeness. In the form, judges responded for each word whether it was familiar, using five response options according to the Likert scale: 1 – Not familiar; 2 – Slightly familiar; 3 – Familiar; 4 – Very familiar; 5 – Extremely familiar, Then, for the same word, they responded regarding its representativeness, that is, whether it could be represented with a drawing or a toy. The response options were:1 – Yes, if it was representative. 2 – No, if it was not representative.

In Tables 1 and 2, a list of words was organized based on the responses of the three groups of expert judges, distributed according to their mean values for familiarity and representativeness.

**Table 1:** Description of words and mean value of the familiarity analysis

**SELECTED WORDS**	**M**
BATATA (potato), PENTE (comb), XÍCARA (cup), PROFESSORA (teacher), MOTO (motorcycle), SOPA (soup), PISCINA (swimming pool), LER (to read), ELEFANTE (elephant), ÓCULOS (glasses), PICOLÉ (popsicle), CHOCOLATE (chocolate), CAMINHÃO (truck), GARRAFA (bottle), AZUL (blue)	2.5
GUARDA-ROUPA (wardrobe), PORCO (pig), SUJO (dirty), GUARDA-CHUVA (umbrella), COBERTOR (blanket), AMARELO (yellow), MORANGO (strawberry), PIA (sink), COMPUTADOR (computer), UMBIGO (belly button), BANHEIRA (bathtub), AREIA (sand), BERÇO (crib), COELHO (rabbit)	2.6
BORBOLETA (butterfly), PIJAMA (pajamas), UVA (grape), PAPEL (paper), LÁPIS (pencil), CHUVEIRO (shower), IRMÃO/IRMÃ (brother/sister), BLUSA (blouse), SALA (living room), FOTO (photo), VASSOURA (broom), VESTIR (to dress), PIPOCA (popcorn), CHINELO (sandal), BOLSA (bag), CARNE (meat), NUVEM (cloud), MACACO (monkey), CASACO (coat)	2.7
AVIÃO (airplane), ESCOVA (brush), CAMISETA (T-shirt), LARANJA (orange), LEÃO (lion), GELADEIRA (refrigerator), CHAPÉU (hat), ORELHA (ear), CHAVE (key), SABONETE (soap), QUARTO (bedroom), SAPO (frog), LÍNGUA (tongue)	2.8
ÁRVORE (tree), JANELA (window), PEDRA (stone), TELEFONE (telephone), MENINA/MENINO (girl/boy), MAÇA (apple), FOGÃO (stove), PASSARINHO (little bird), BICICLETA (bicycle), BOLO (cake), TOALHA (towel), PEIXE (fish), COMIDA (food), PENTEAR (to comb), ESCORREGADOR (slide)	2.9
CALÇA (pants), COZINHA (kitchen), PATO (duck), BALANÇO (swing), LIVRO (book), SAPATO (shoe), LEITE (milk), DOENTE/DODÓI (sick/hurting), ESTRELA (star), CADEIRA (chair), PORTA (door), TRAVESSEIRO (pillow), VACA (cow), MESA (table), ESCOVA DE DENTE (toothbrush), CAVALO (horse), LUA (moon), FACA (knife)	3.0
GALINHA (chicken), DENTE (tooth), PÃO (bread), SOFÁ (sofa), FEIJÃO (bean), BANHEIRO (bathroom), GARFO (fork), OVO (egg), BOLACHA/BISCOITO (cookie), BALÃO (balloon), ROUPA (clothes), PRATO (plate), COMIDA (food), MEIA (sock), NENÉM (baby)	3.1
PERNA (leg), FLOR (flower), TELEVISÃO (TV) (television), AVÔ/AVÓ (grandfather/grandmother), BUMBUM (bottom), DEDO (finger), CHUPETA (pacifier), COCÔ (poop), SUCO (juice), COLHER (spoon), DORMIR (to sleep)	3.2
ARROZ (rice), SOL (sun), CAMA (bed), OLHO (eye), FRALDA (diaper), BONECA (doll), CABELO (hair), BANANA (banana), COPO (glass), COMER (to eat), BRAÇO (arm), MÃO (hand), NARIZ (nose), MAMADEIRA (baby bottle), GATO (cat), CABEÇA (head)	3.3
CARRO (car), PAI/PAPAI (dad), CACHORRO (dog), CASA (house), BARRIGA (belly), PÉ (foot)	3.4
BOLA (ball), BOCA (mouth), ÁGUA (water), MÃE/MAMÃE (mom)	3.5

**Table 2:** Description of the words and mean value of the representativeness analysis

**Words**	**M**
AREIA (sand), NUVEM (cloud), ROUPA (clothes), SALA (living room), FOTO (photo), VESTIR (to dress), CARNE (meat), QUARTO (bedroom), JANELA (window), PEDRA (stone), COMIDA (food), ARROZ (rice), ÁGUA (water), SOPA (soup), LER (to read), SUJO (dirty), IRMÃO/IRMÃ (brother/sister), COMIDA (food), PENTEAR (to comb), COZINHA (kitchen), BANHEIRO (bathroom), COCÔ (poop), COMER (to eat).	0.8
PENTE (comb), PROFESSORA (teacher), PISCINA (swimming pool), ELEFANTE (elephant), PICOLÉ (popsicle), AZUL (blue), GUARDA-ROUPA (wardrobe), COBERTOR (blanket), AMARELO (yellow), PIA (sink), BERÇO (crib), BORBOLETA (butterfly), PIJAMA (pajamas), PAPEL (paper), LÁPIS (pencil), CHUVEIRO (shower), BLUSA (blouse), PIPOCA (popcorn), CHINELO (sandal), CASACO (coat), CAMISETA (T-shirt), CHAVE (key), SABONETE (soap), LÍNGUA (tongue), TOALHA (towel), ESCORREGADOR (slide), CALÇA (pants), BALANÇO (swing), ESTRELA (star), CADEIRA (chair), TRAVESSEIRO (pillow), ESCOVA DE DENTE (toothbrush), LUA (moon), FLOR (flower), DORMIR (to sleep), SOL (sun), FRALDA (diaper), AVÔ/AVÓ (grandfather/grandmother), PAI/PAPAI (dad), MÃE/MAMÃE (mom).	0.9
BATATA (potato), XÍCARA (cup), MOTO (motorcycle), ÓCULOS (glasses), CHOCOLATE (chocolate), CAMINHÃO (truck), GARRAFA (bottle), PORCO (pig), GUARDA-CHUVA (umbrella), MORANGO (strawberry), COMPUTADOR (computer), UMBIGO (belly button), BANHEIRA (bathtub), COELHO (rabbit), UVA (grape), VASSOURA (broom), BOLSA (pocket), MACACO (monkey), AVIÃO (airplane), ESCOVA (brush), LARANJA (orange), LEÃO (lion), GELADEIRA (refrigerator), CHAPÉU (hat), ORELHA (ear), SAPO (frog), ÁRVORE (tree), TELEFONE (telephone), MENINA/MENINO (girl/boy), MAÇA (apple), FOGÃO (stove), PASSARINHO (little bird), BICICLETA (bicycle), BOLO (cake), PEIXE (fish), PATO (duck), LIVRO (book), SAPATO (shoe), LEITE (milk), DOENTE/DODÓI (sick/hurting), PORTA (door), VACA (cow), MESA (table), CAVALO (horse), FACA (knife), GALINHA (chicken), DENTE (tooth), PÃO (bread), SOFÁ (sofa), FEIJÃO (bean), GARFO (fork), OVO (egg), BOLACHA/BISCOITO (cookie), BALÃO (balloon), PRATO (plate), MEIA (sock), NENÉM (baby), PERNA (leg), TELEVISÃO (TV) (television), BUMBUM (bottom), DEDO (finger), BICO/CHUPETA (pacifier), SUCO (juice), COLHER (spoon), CAMA (bed), OLHO (eye), BONECA (doll), CABELO (hair), BANANA (banana), COPO (glass), BRAÇO (arm), MÃO (hand), NARIZ (nose), MAMADEIRA (baby bottle), GATO (cat), CABEÇA (head), CARRO (car), CACHORRO (dog), CASA (house), BARRIGA (belly), PÉ (foot), BOLA (ball), BOCA (mouth).	1.0

### Data analysis

According to the judges’ responses, in order to generate the word list that would make up the word bank, a descriptive analysis was conducted through the distribution of the mean of means of the 480 words, taking into account the value of the mean of means in terms of familiarity and representativeness. Words that did not fall within this range were discarded.

The analysis of the data resulting from this research was quantitative, descriptive, and inferential. The data were analyzed using the R software (R Core Team), specifically with the help of packages designed for text data mining (SnowballC, tm, and wordcloud2). The words presented by the three groups of judges were displayed in frequency tables and also through the word cloud illustration technique generated by the software. The most frequently used familiar and representative words are highlighted in the cloud according to position, color, and size.

They were organized as follows:

● Word **size** is determined by **representativeness**. That is, the larger the word appears in the cloud, the more representative it is considered. Large words indicate central, typical, or defining concepts of the context. Small words are less representative, perhaps more peripheral or neutral.

● Word **color** represents **familiarity**. It varies along a continuous gradient (from dark blue to yellow/white). The lighter the color is (for example, yellowish green), the greater the familiarity it indicates. Darker colors (for example, purple/dark blue) indicate low familiarity.

## RESULTS

The groups of judges who participated in the consolidation of this word bank were divided according to the number of responses provided, since not all judges answered both questions. As a result, they were organized as follows: 21 judges for familiarity, consisting of 9 speech-language pathologists who were experts in the field, 6 speech-language pathologists who were mothers of children, and 6 mothers who were not speech-language pathologists. For representativeness, there were 13 judges in total, consisting of 7 speech-language pathologists who were experts in the field, 3 speech-language pathologists who were mothers of children, and 3 mothers who were not speech-language pathologists.

According to the criteria for selecting the words that made up the new word bank, only those meeting the values derived from the mean of means were selected. That is, based on the judges’ responses, only the words considered familiar with a mean value greater than 2.5 and representative with a mean value greater than 0.2 were included.

**Figure 1:**
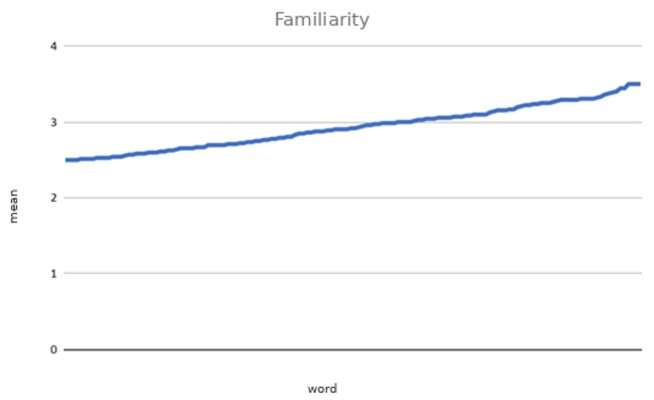
Distribution curve of the mean values for familiarity

**Figure 2:**
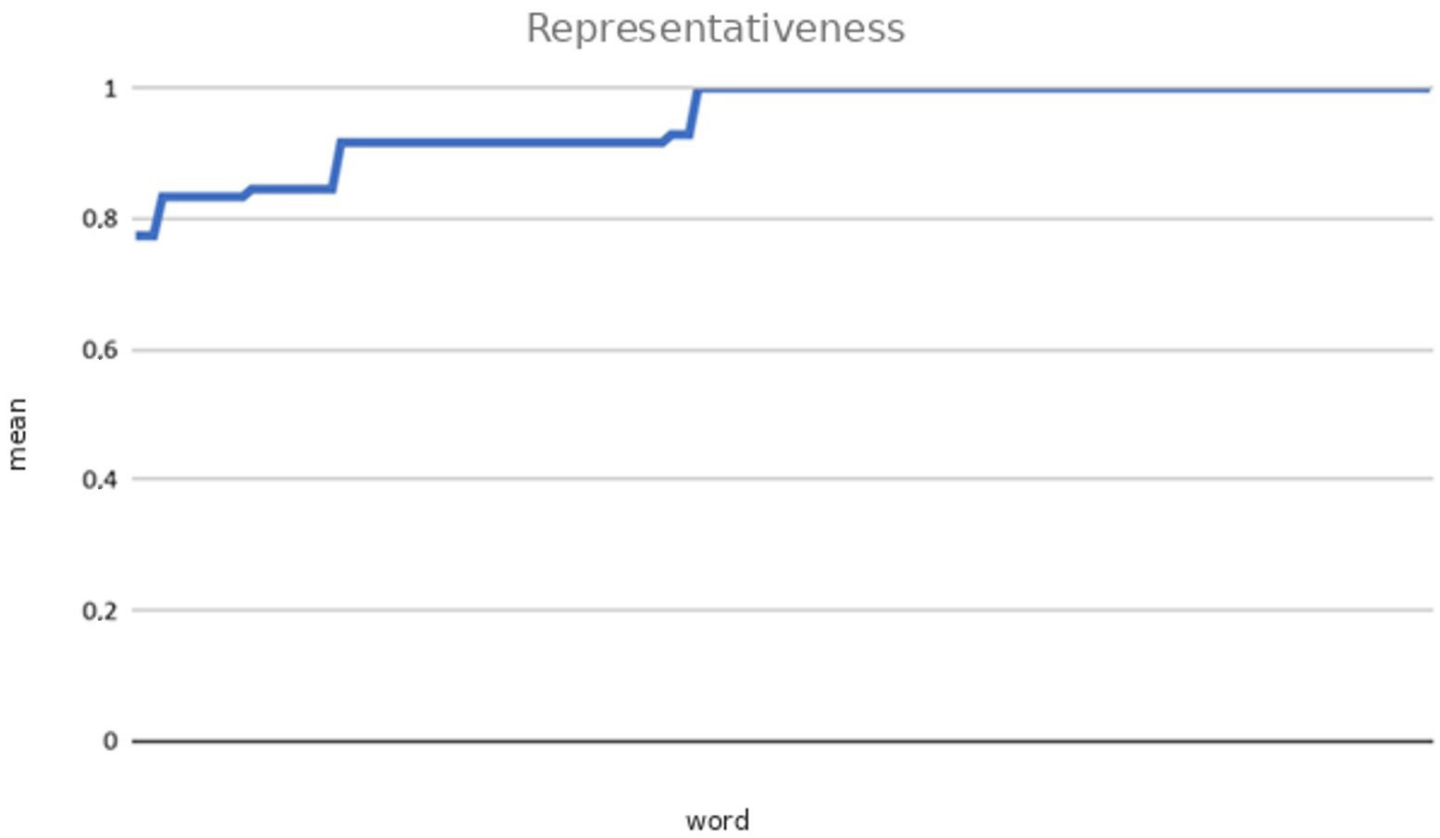
Distribution curve of the mean values for representativeness

The results in Figure 1 represent the selection criterion for words whose mean value was above 2.5, while Figure 2 represents the selection criterion for words whose mean value was above 0.2.

The words are presented in Brazilian Portuguese, since the collection, evaluation, and analysis were conducted with a child population speaking this language, thereby preserving the cultural and linguistic validity of the material.

The total number of words selected based on the judges’ responses was 146, according to the mean values for both familiarity and representativeness. In the tables, they are distributed according to familiarity and representativeness.

**Figure 3:**
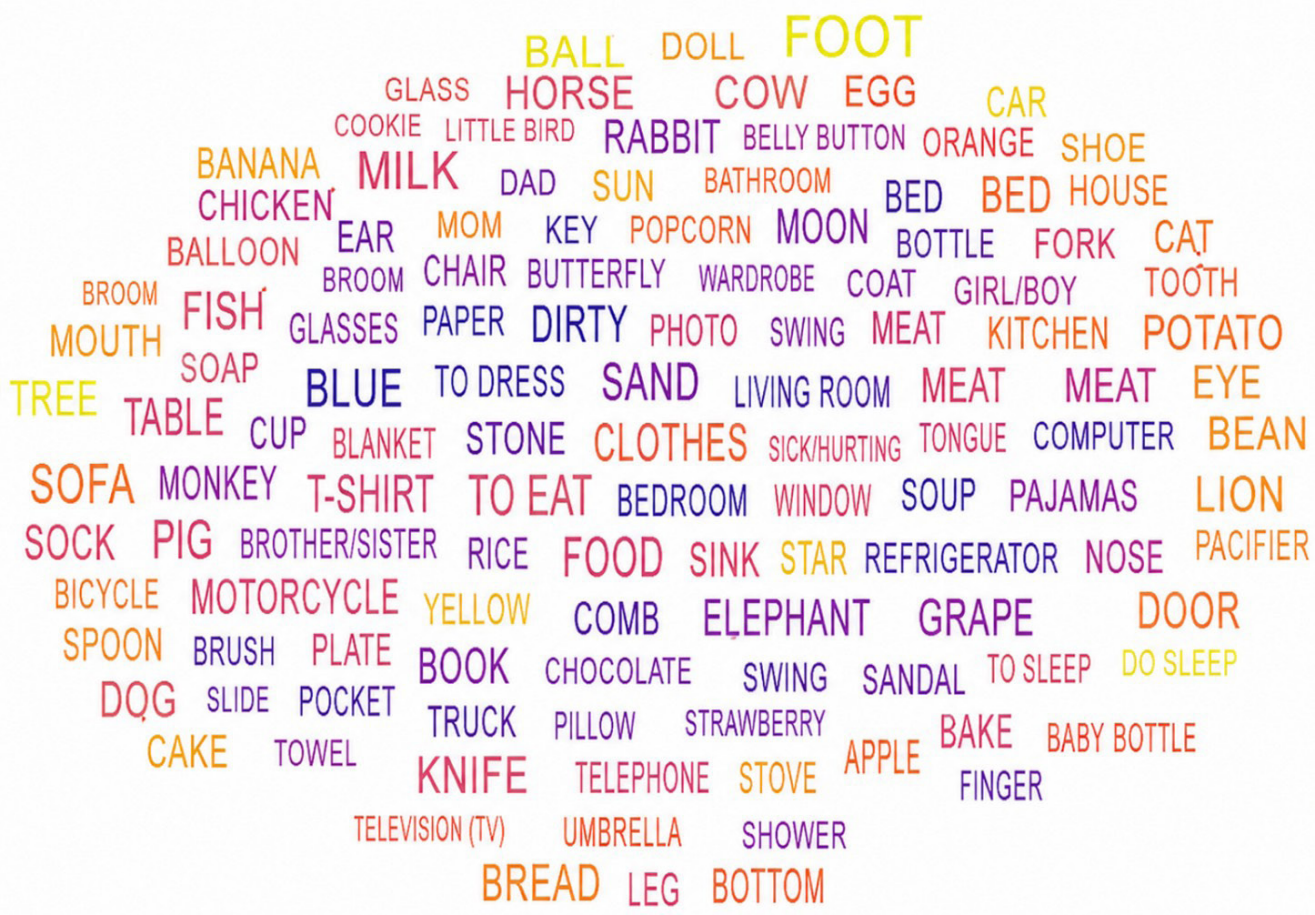
Distribution of familiar and representative words

The familiar and representative words were distributed as follows:

Table 1 presents the list of words and the mean value for familiarity, whereas Table 2 lists the words and the mean value for representativeness. In Figure 3, the words are represented by colors and sizes, with larger size indicating greater representativeness and lighter color indicating greater familiarity.

First, words with high representativeness and familiarity are identified, recognizable by their large size and light colors, close to yellow. Within this category, and according to their semantic grouping, the following are included: in body parts: pé (foot), mão (hand), boca (mouth), and olho (eye); in animals: gato (cat) and pato (duck); in objects and utensils: copo (cup), pente (comb), and bola (ball); in foods: ovo (egg) and suco (juice); and in environmental elements: sol (sun) and casa (house). These words occupy central positions in the cloud and present high visibility.

Second, terms with high representativeness but lower familiarity are observed, characterized by large size and darker colors, with violet or bluish shades. Among them are, in foods: uva (grape) and maçã (apple); in animals: leão (lion); in objects: livro (book) and faca (knife); and in actions or concepts: ler (to read). Although they maintain a prominent position in the overall arrangement, their color indicates a lower degree of familiarity reported by the judges.

Third, words with lower representativeness but high familiarity appear, identified by their small size and light tone. These include, in foods: banana (banana), bolo (cake), and arroz (rice); in animals: cachorro (dog) and galinha (chicken); in objects: cama (bed) and prato (plate). These terms are mostly distributed on the periphery of the cloud, while still maintaining coloration indicative of high familiarity.

Finally, words with low representativeness and low familiarity are distinguished, combining small size and dark colors. These include, in objects and utensils: escorregador (slide), travesseiro (pillow), and guarda-chuva (umbrella); in actions: pentear (to comb) and vestir (to dress); and in concepts or child expressions: dodói (owie/pain) and bumbum (bottom). Their presence is smaller, and their peripheral location reflects their limited visual prominence in the cloud.

## DISCUSSION

The validation process using both expert judges and caregivers, in this case mothers with or without knowledge in the field of speech-language pathology, is essential to ensure that the selected words are relevant from both a technical and a contextual perspective.

Combining the perceptions of experienced speech-language pathologists, mothers with a background in speech-language pathology, and mothers without such training generates strong content validity that reflects the actual use of child language.

This practice is recommended in cross-cultural validations of assessment instruments, as evidenced in studies such as PEEPS-BP, where similar methods were successfully employed to ensure representativeness and semantic fidelity in the Brazilian context^(7)^_._

Studies have shown that the characteristics of child-directed communication (Comunicação Dirigida para a Criança - CDC) support language acquisition in both comprehension and production. Therefore, these new child-oriented word association standards provide a novel perspective on the semantic context of young children and early lexical development^(12)^.

Including criteria such as selecting words based on familiarity and representativeness optimizes the quality of the word bank for a child. Lexical familiarity has been shown to be a robust predictor of early language development, including expressive vocabulary and phonology in early ages^(13)^.

Including mothers makes it possible to capture cultural and semantic aspects that are not always evident in the technical literature. Research in early intervention has shown that the convergence between the professional perspective and the maternal perspective improves family adherence and treatment effectiveness. Studies on early lexical development reveal that word familiarity predicts acquisition better than variables such as frequency or word length^(14)^.

At the same time, focusing on words with high familiarity and low representativeness ensures that the bank is accessible while also being discriminative for the age group. This explains the results regarding words that are normally associated with household elements and places.

In Brazil, there are several validated inventories that measure children’s lexical repertoires, such as studies on vocabulary at 24 and 30 months based on the MacArthur Inventory and others developed locally^(4,6-8)^. However, most lack an item selection process based on familiarity and representativeness criteria validated by expert judges and mothers with experience in the field.

Although the applied criteria delimited 146 words, the inclusion of familiar and representative words outside the thresholds could broaden the assessment range. The creation of this word bank, based on expert judgment and solid quantitative criteria, represents a practical and scientifically grounded tool^(15)^.

It not only promotes an assessment adapted to the Brazilian population, but also fosters better clinical practices in early speech-language pathology. In this way, the resulting 146 words align with the appropriate age ranges and ensure that each term is known and useful for clinical assessment. The designed instrument allows speech-language pathologists to assess quickly and specifically the lexical repertoire of young children.

Vocabulary selection should be anchored in a bioecological approach that incorporates the child’s chronological age, cultural context, and family environment. Bronfenbrenner posits that the dynamic interaction between internal and environmental factors shapes language development^(16)^.

Contemporary studies in South African and Canadian children confirm that the type of language input, sometimes measured through verbal interaction at home, bilingual exposure, or shared reading practices, is a robust predictor of the breadth and depth of receptive and expressive vocabulary^(17,18)^.

Recent studies show that both the frequency and the accuracy of a word increase its likelihood of being understood and produced by children between 16 and 30 months, with synergistic interactions that decline with age^(19)^. A meta-analysis on age of acquisition indicates that terms acquired at an early age are processed more quickly and accurately^(20)^.

This new bank of 146 words fills that gap (Appendix A), offering an instrument with evidence of content validity, applied in clinical stages and supported by statistical analyses. This emphasizes that the words included in assessments should reflect both passive knowledge and the ability for active use in real communicative contexts, promoting the functional relationship of language across family, school, and cultural settings^(21)^.

Recent studies indicate that phonological tools adapted to Brazil’s linguistic and cultural context favor more precise and effective outcomes in speech-language intervention. In addition, having a reliable and validated list contributes to institutional and professional consistency, promoting evidence-based clinical practice.

## CONCLUSION

This research study provides a tool for the clinical use of health professionals. A total of 146 available words were identified and considered familiar and representative. This word bank is not only useful for assessing early language, but also has broad therapeutic potential. The selected words may serve as stimuli for phonological, narrative, and augmentative and alternative communication (AAC) assessment and intervention strategies.

## Appendix A Word Bank for Child Language Development (BPDLI): A Validated Instrument for Brazilian Portuguese

Preamble

This instrument is a practical, functional, and culturally validated tool, specifically designed for Speech-Language Pathology professionals who work with the Brazilian population. The BPDLI emerged as the result of a rigorous research process that included the collection and validation of a set of words that are highly representative and familiar for young Brazilian Portuguese-speaking children. The words included in this bank have been organized into categories, following the structure of the well-known MacArthur Communicative Development Inventory, adapted to the linguistic and cultural particularities of Brazil. (See Table 3).

**Table 3:** Word Bank for Child Language Development (BPDLI): Validated Instrument for Brazilian Portuguese

**WORD**	**FAMILIARITY**	**REPRESENTATIVENESS**	**CATEGORY**
TO EAT	3.292	0.845	**ACTION WORDS**
TO SLEEP	3.236	0.917	
TO READ	2.514	0.845	
TO COMB	2.931	0.845	
TO DRESS	2.694	0.833	
BUTTERFLY	2.653	0.917	**ANIMALS**
DOG	3.389	1.000	
HORSE	3.042	1.000	
RABBIT	2.639	1.000	
ELEPHANT	2.528	0.917	
CHICKEN	3.056	1.000	
CAT	3.319	1.000	
LION	2.778	1.000	
MONKEY	2.736	1.000	
LITTLE BIRD	2.903	1.000	
DUCK	2.972	1.000	
FISH	2.917	1.000	
PIG	2.569	1.000	
FROG	2.847	1.000	
COW	3.014	1.000	
WATER	3.500	0.833	**FOODS AND DRINKS**
RICE	3.250	0.833	
BANANA	3.292	1.000	
POTATO	2.500	1.000	
COOKIE	3.083	1.000	
CAKE	2.903	1.000	
MEAT	2.722	0.833	
CHOCOLATE	2.528	1.000	
FOOD	2.917	0.845	
BEAN	3.069	1.000	
MILK	2.986	1.000	
APPLE	2.889	1.000	
STRAWBERRY	2.597	1.000	
EGG	3.083	1.000	
BREAD	3.056	1.000	
POPSICLE	2.528	0.917	
POPCORN	2.708	0.917	
SOUP	2.514	0.845	
JUICE	3.222	1.000	
GRAPE	2.653	1.000	
YELLOW	2.583	0.917	**QUALITIES AND ATTRIBUTES**
BLUE	2.542	0.917	
SICK/ HURTING	2.986	1.000	
ORANGE	2.764	1.000	
DIRTY	2.569	0.845	
BALLOON	3.097	1.000	**TOYS**
BALL	3.500	1.000	
DOLL	3.292	1.000	
PENCIL	2.667	0.917	
BOOK	2.986	1.000	
HOUSE	3.403	1.000	**PLACES OUTSIDE THE HOME**
SWIMMING POOL	2.514	0.917	
BATHTUB	2.611	1.000	**FURNITURE AND ROOMS**
BATHROOM	3.069	0.845	
CRIB	2.625	0.917	
CHAIR	3.000	0.917	
SHOWER	2.667	0.917	
KITCHEN	2.958	0.845	
STOVE	2.889	1.000	
REFRIGERATOR	2.778	1.000	
WARDROBE	2.556	0.917	
WINDOW	2.861	0.833	
TABLE	3.028	1.000	
SINK	2.597	0.917	
DOOR	3.000	1.000	
BEDROOM	2.833	0.833	
LIVING ROOM	2.694	0.833	
SOFA	3.056	1.000	
TELEVISION	3.153	1.000	
SAND	2.625	0.774	**OUTDOOR OBJECTS**
TREE	2.861	1.000	
SWING	2.972	0.917	
SLIDE	2.944	0.917	
STAR	3.000	0.917	
FLOWER	3.153	0.917	
MOON	3.042	0.917	
CLOUD	2.722	0.774	
STONE	2.875	0.833	
SUN	3.250	0.917	
BELLY	3.444	1.000	**BODY PARTS**
PACIFIER	3.208	1.000	
MOUTH	3.500	1.000	
ARM	3.306	1.000	
BOTTOM	3.167	1.000	
HEAD	3.333	1.000	
HAIR	3.292	1.000	
POOP	3.222	0.845	
FINGER	3.194	1.000	
TOOTH	3.056	1.000	
TONGUE	2.847	0.917	
HAND	3.306	1.000	
NOSE	3.306	1.000	
EYE	3.264	1.000	
EAR	2.792	1.000	
FOOT	3.444	1.000	
LEG	3.153	1.000	
BELLY BUTTON	2.611	1.000	
GRANDFATHER/GRANDMOTHER	3.167	0.929	**PEOPLE**
BROTHER/SISTER	2.667	0.845	
MOM	3.500	0.929	
GIRL/BOY	2.875	1.000	
BABY	3.139	1.000	
DAD	3.375	0.929	
TEACHER	2.500	0.917	
BLOUSE	2.694	0.917	**CLOTHING AND ACCESSORIES**
BAG	2.708	1.000	
PANTS	2.958	0.917	
T-SHIRT	2.764	0.917	
COAT	2.736	0.917	
HAT	2.792	1.000	
SANDAL	2.708	0.917	
DIAPER	3.278	0.917	
UMBRELLA	2.583	1.000	
SOCK	3.125	1.000	
GLASSES	2.528	1.000	
PAJAMAS	2.653	0.917	
CLOTHES	3.097	0.774	
SHOE	2.986	1.000	
FOOD	3.097	0.833	**DAILY ROUTINE**
BED	3.250	1.000	**HOUSEHOLD ITEMS**
KEY	2.806	0.917	
BLANKET	2.583	0.917	
SPOON	3.236	1.000	
COMPUTER	2.597	1.000	
GLASS	3.292	1.000	
BRUSH	2.750	1.000	
TOOTHBRUSH	3.028	0.917	
KNIFE	3.042	1.000	
PHOTO	2.694	0.833	
FORK	3.069	1.000	
BOTTLE	2.542	1.000	
BABY BOTTLE	3.306	1.000	
PAPER	2.653	0.917	
COMB	2.500	0.917	
PLATE	3.097	1.000	
SOAP	2.806	0.917	
TELEPHONE	2.875	1.000	
TOWEL	2.903	0.917	
PILLOW	3,.000	0.917	
BROOM	2.694	1.000	
CUP	2.500	1.000	
AIRPLANE	2.750	1.000	**VEHICLES**
BICYCLE	2.903	1.000	
TRUCK	2.542	1.000	
CAR	3.361	1.000	
MOTORCYCLE	2.514	1.000	

Objective

The main objective of the BPDLI is to provide speech-language pathologists with a solid clinical and functional foundation to optimize intervention planning, monitor progress in language and speech development, and select relevant vocabulary during the therapeutic process.

Instructions for Use

The BPDLI was conceived as a fundamental reference guide for the assessment of expressive language in young children. For its application, the speech-language pathologist may use the word list to identify those words that the child recognizes as familiar and those that are representative of the child’s active linguistic repertoire. This information is crucial for establishing individualized therapeutic goals and designing intervention strategies tailored to the specific needs of each child.

## References

[1] Capovilla F, Alessandra S (1997). Desenvolvimento linguístico na criança brasileira dos dois aos seis anos: Tradução e estandardização do Peabody Picture Vocabulary Test de Dunn & Dunn e da Language Development Survey de Rescorla. Ciênc Cogn.

[2] Graham L, Graham A, West C (2015). From research to practice: the effect of multi-component vocabulary instruction on increasing vocabulary and comprehension performance in social studies. Int Electron J Elem Educ.

[3] Wright TS, Cervetti GN (2016). A systematic review of the research on vocabulary instruction that impacts text comprehension. Read Res Q.

[4] Savoldi A, Barichello Gubiani M, Brancalioni AR, Keske-Soares M (2012). Relação entre as palavras eliciadas na Avaliação Fonológica da Criança e as variáveis idade, gênero e gravidade do desvio fonológico. Audiol Commun Res.

[5] Suárez Palacio PA, Vélez Múnera M (2018). El papel de la familia en el desarrollo social del niño: una mirada desde la afectividad, la comunicación familiar y estilos de educación parental. Psicoespacios..

[6] Oshima M, Moret ALM, Amorim RB, Alvarenga KDF, Bevilacqua MC, Lauris JRP (2010). Early Listening Function (ELF): adaptação para a língua portuguesa. Rev Soc Bras Fonoaudiol.

[7] Simoni SN, Morares DA, Pagliarin KC, Keske-Soares M (2024). Validade de conteúdo do Profiles of Early Expressive Phonological Skills-Brazilian Portuguese (PEEPS-BP) - Lista expandida. CoDAS.

[8] Barbosa ALDA, Soares HB, Azoni CAS (2019). Construção de um instrumento de triagem do vocabulário para crianças entre 3 e 7 anos. Audiol Commun Res.

[9] Wright TS, Cervetti GN (2017). A systematic review of the research on vocabulary instruction that impacts text comprehension. Read Res Q.

[10] Elleman AM, Oslund EL, Griffin NM, Myers KE (2019). A review of middle school vocabulary interventions: five research-based recommendations for practice. Lang Speech Hear Serv Sch.

[11] Teixeira ER (2005). CDI’s adaptation to Brazilian portuguese: validation study of the words and sentences form..

[12] Cox CR, Haebig E (2023). Child-oriented word associations improve models of early word learning. Behav Res Methods.

[13] Munro N, Baker E, Masso S, Carson L, Lee T, Wong AM (2021). Vocabulary acquisition and usage for late talkers treatment: effect on expressive vocabulary and phonology. J Speech Lang Hear Res.

[14] Braza MD, Porter HL, Buss E, Calandruccio L, McCreery RW, Leibold LJ (2022). Effects of word familiarity and receptive vocabulary size on speech-in-noise recognition among young adults with normal hearing. PLoS One.

[15] Kempe V, Ota M, Schaeffler S (2024). Does child-directed speech facilitate language development in all domains? A study space analysis of the existing evidence. Dev Rev.

[16] Bargetto Fernández MÁ, Riffo Ocares B (2019). El reconocimiento de palabras y el acceso léxico: revisión de modelos y pruebas experimentales. Bol Filol.

[17] Southwood F, White MJ, Brookes H, Pascoe M, Ndhambi M, Yalala S (2021). Sociocultural factors affecting vocabulary development in young south african children. Front Psychol.

[18] Dodorico L, Carubbi S, Salerni N, Calvo V (2001). Vocabulary development in Italian children: a longitudinal evaluation of quantitative and qualitative aspects. J Child Lang.

[19] Miller LM, Roodenrys S (2009). The interaction of word frequency and concreteness in immediate serial recall. Mem Cognit.

[20] Elsherif MM, Preece E, Catling JC (2023). Age-of-acquisition effects: A literature review. J Exp Psychol Learn Mem Cogn.

[21] Bornstein MH, Haynes OM (1998). Vocabulary competence in early childhood: measurement, latent construct, and predictive validity. Child Dev.

