# Supplementary material for: Words for clinical use in speech-language pathology in Brazilian portuguese-speaking children: a focus on familiarity and representativeness from expert judges
Source: Codas. 2026 Jul 31;38(5):e20250307. doi: 10.1590/2317-1782/e20250307en (PMC13440680; doi:10.1590/2317-1782/e20250307en)
Supplement: Translation

# Translation: Palabras para uso clínico fonoaudiológico en niños hablantes de portugués brasilero: enfoque desde la familiaridad y representatividad de jueces expertos

**DOI:** 10.1590/2317-1782/e20250307es

**Authors:** Yanela Paola Jaimes Parada, Vitor Cantele Malavolta, Denis Altieri de Oliveira Moraes, Simone Nicolini de Simoni, Márcia Keske-Soares

**Affiliations:** 1 Programa de Pós-graduação em Distúrbios da Comunicação Humana, Universidade Federal de Santa Maria – UFSM - Santa Maria (RS), Brasil.

**Keywords:** es: Desarrollo del Lenguaje, Lenguaje Infantil, Vocabulario, Pruebas del Lenguaje, Estudio de Validación

## Abstract

**Objetivo:**

Proporcionar un banco de palabras basado en la familiaridad y representatividad de las palabras para el intervalo de 18 a 36 meses, considerando el contexto cultural brasileño.

**Método:**

Se trata de una investigación transversal con un diseño cuantitativo. El cuestionario, enviado a los jueces especialistas, se basa en la lista de palabras y oraciones del Inventario de Desarrollo de la Comunicación (The MacArthur-Bates Communicative Development Inventories - MB-CDIs). Participaron tres grupos de expertos: logopedas expertos, logopedas y madres sin formación en el área. El cuestionario incluía 21 preguntas de familiaridad y 13 de representación, que se respondían mediante una escala Likert sobre familiaridad (familiar o desconocido) y representatividad (representación por imágenes o no). Las respuestas fueron analizadas utilizando estadística descriptiva e inferencial y representadas visualmente a través de nubes de palabras.

**Resultados:**

De acuerdo con los criterios, se seleccionaron solo aquellas que cumplieran con los valores de media, quedando un total de 146 palabras en total. A partir de la lista del inventario MB-CDIs adaptado al portugués, se organizaron por categorías para generar el banco de palabras, llamado Banco de Palabras para el Desarrollo Lingüístico Infantil (BPDLI): un Instrumento Validado para Portugués Brasileño.

**Conclusión:**

El trabajo aporta una herramienta validada que puede ser utilizada en la evaluación e intervención clínica del lenguaje en edades tempranas, permitiendo una mayor sensibilidad al contexto cultural y familiar del niño. Este banco de palabras también posee potencial terapéutico para enfoques en fonología, lenguaje narrativo y comunicación aumentativa y alternativa.

## INTRODUCCIÓN

El desarrollo neurobiológico del ser humano influye sobre los procesos del lenguaje verbal. Desde los aspectos sensoriales, principalmente desde la estimulación auditiva, desde que se nace se está expuesto a estímulos sonoros que son el punto de partida para la adquisición del repertorio verbal de cualquier niño^(1)^_._

Además, que, durante los primeros años de vida, este se da a partir de las experiencias que vive dentro de su propio contexto, la interacción le permite que a mayores vivencias represente más crecimiento lexical^(2)^_._

Se ha mencionado esta conexión en la literatura científica, a través de redes que traen consigo unos significados asociados a unos nuevos aprendizajes. Aunque muchos logran alcanzar el desarrollo del lenguaje adecuadamente para la edad, para otros representa desafíos^(3)^_._

Si bien es cierto que desde antes de nacer se cuenta con los prerrequisitos indispensables para desarrollar un sistema comunicativo, durante la niñez se alcanza la mayor captación de estímulos fonológicos que luego serán los encargados de contribuir secuencialmente a muchos procesos funcionales para el desarrollo del habla^(4)^_._

Aprender nuevas palabras es directamente proporcional a las experiencias que como familia se viven y terminan siendo el punto de partida para un amplio vocabulario rico en producciones. Ya que estas son la primera fuente de significado que con el tiempo experimentará nuevos contextos y por ende nuevos retos de acuerdo con el ambiente definido en este estudio como proceso simbólico transaccional^(3,5).^

Actualmente se ha venido evidenciando el incremento del interés en crear herramientas en todas las áreas fonoaudiológicas, que apoyen el uso en los profesionales que se focalizan en los aspectos normales y atípicos que influyen en el lenguaje. Siendo preciso involucrar también a las familias ya que son las primeras en detectar si existe alguna anomalía.

En el área de audiología se hizo un estudio en Brasil sobre la traducción y validación cultural de un instrumento *Early Listening Function* (ELF)^(6)^_._ Diseñado para aplicar en casa como proceso inicial en la evaluación auditiva. Este aporte permite incorporar a la familia como ente receptivo en la detección precoz de conductas auditivas y así involucra a los padres estén atentos ante situaciones del hogar.

Por otro lado, se logra evidenciar que, dentro de la evaluación fonoaudiológica en el área de habla, se continúa ampliando la importancia de extender los estudios sobre el componente fonológico.

Es primordial que un repertorio fonológico sea lo suficientemente amplio para que tenga en cuenta todos los sonidos del habla según como lo demuestra un estudio realizado a partir de la búsqueda de la relación entre las palabras que pertenecen y no hacen parte de la evaluación fonológica en niños (*Avaliação Fonológica da Criança* - AFC) en este trabajo destaca la evaluación fonológica de niños teniendo en cuenta el género y la edad^(4)^_._

Otro aporte importante para la tipificación precoz de niños con posibles dificultades del habla en Brasil, fue el realizado a partir de un instrumento internacional, *Profiles of Early Expressive Phonological Skills- Brazilian Portuguese* (PEEPS-BP), el cual fue traducido y validado transculturalmente contribuyendo a la selección de instrumentos de acuerdo con la población brasileña^(7)^_._

Para la construcción de instrumentos para evaluar el vocabulario infantil, seleccionar palabras se considera su familiaridad y relevancia cultural. Por ejemplo, en la creación de una herramienta de evaluación para niños de 3 a 7 años, se eligieron palabras comunes en diferentes regiones y contextos culturales de Brasil, asegurando que fueran representativas y fácilmente reconocibles por los niños^(8)^_._

Teniendo en cuenta las diversas y amplias variables, como los demuestran algunos de los estudios realizados en el campo fonológico en Brasil^(4,6-8)^ se han aumentado el interés en mejorar las herramientas de evaluación sobre todo en edades tempranas y teniendo en cuenta el vocabulario y la fonología propias para la población a la cual se aplica. Estos establecen la importancia de saber seleccionar las palabras, ya que esto determina la eficacia en lo que se desea realizar generando beneficios a partir de los mapas semánticos entre otras variables que permitan que la creación de palabras no sea de manera superficial^(9,10)^_._

Además, los estudios han demostrado que el desempeño de niños en tareas de percepción del habla mejora significativamente cuando se utilizan listas de palabras familiares para ellos, en comparación con listas estandarizadas^(4)^_._ Esto resalta la importancia de considerar la familiaridad y representatividad de las palabras en evaluaciones fonoaudiológicas.

Este trabajo tiene como objetivo proporcionar un banco de palabras basado en la familiaridad y representatividad de las palabras para el intervalo de 18 a 36 meses.

## MÉTODOS

Este estudio se caracteriza por ser una investigación transversal, con diseño cuantitativo, que contó con la participación de jueces expertos y no expertos para la construcción de un Banco de Palabras para el Desarrollo Lingüístico Infantil (BPDLI): un Instrumento Validado para Portugués Brasileño. El BPDLI se basó en la familiaridad y frecuencia de las palabras producidas por niños de 18 a 36 meses, según los informes de los cuidadores. El estudio es de naturaleza observacional y fue escrito de acuerdo con la Declaración STROBE.

Esta investigación cumple con los requisitos exigidos ya que fue aprobada por el comité de ética (CEP) de una institución de educación superior identificada con el número 3.505.454. Además, los participantes firmaron los consentimientos informados para autorización de esta. La investigación fue realizada con base en la resolución 466/12 teniendo en cuenta los requisitos para la protección de los datos.

### Participantes

Conformado por 3 tipos de jueces expertos y no expertos para un total de 21 jueces. Dentro de los criterios para la selección del grupo de jueces, se tuvo en cuenta que pertenecieran a nivel nacional y estuvieran en cualquiera de los estados de Brasil y cuyo desempeño profesional fuera en el área de habla y lenguaje y por supuesto su experiencia con niños en fase etaria de 18 a 36 meses. Quedando organizadas de la siguiente forma:

- *Grupo 1 - jueces expertos*: Especialistas en el área de Fonoaudiología. Con título de Doctores y con una experiencia mínima de 5 años en la profesión.
- *Grupo 2* - *madres fonoaudiólogas:* este grupo fue conformado por profesionales en Fonoaudiología que además tenían experiencia de ser madres de niños en edades comprendidas entre 18 y 36 meses.
- *Grupo 3 - madres no fonoaudiólogas:* Este grupo estaba conformado por madres de niños en edades entre los 18 a los 36 meses que no tenían ni conocimientos ni trayectoria en el área de Fonoaudiología.

### Materiales

El cuestionario enviado a los jueces especialistas se basó en la lista de palabras del inventario de desarrollo comunicativo (CDI´s) palabras y oraciones. Versión autorizada del MacArthur-Bates Comunicativa versión brasileña - PARTE I - Uso de palabras - Lista de vocabulario^(11)^. El cual fue construido a modo de pregunta para que cada juez seleccionara de acuerdo con su criterio. Este cuestionario electrónico quedó conformado por un total de 480 palabras.

### Procedimientos

Para llevar a cabo la recolección de los datos se contactaron los tres grupos de jueces a quienes se les envió al correo electrónico, los formularios creados en *Google Docs* invitándolos a ser jueces expertos y contribuir a la selección de palabras de familiaridad y representatividad. En este, los jueces responden, para cada palabra si esta era familiar, con cinco opciones de respuesta: 1 -No familiar.; 2 -Poco familiar; 3 -Familiar; 4 -Muy familiar; 5 - Extremadamente familiar, de acuerdo con la escala Likert. Luego, para la misma palabra, respondía respecto de su representatividad, es decir, si podría ser representada con un dibujo o un juguete. Las opciones de respuesta eran: 1-Si para ser representativa y 2-No, si no era representativa.

En las Tablas 1 y 2, fue organizada una lista de palabras con base a las respuestas de los tres grupos de jueces expertos distribuidas con su valor de media para familiaridad y representatividad.

**Tabla 1:** Descripción de palabras y valor medio del análisis de familiaridad

**PALABRAS SELECCIONADAS**	**M**
BATATA (papa), PENTE (peine), XÍCARA (taza), PROFESSORA (profesora), MOTO (moto), SOPA (sopa), PISCINA (piscina), LER (leer), ELEFANTE (elefante), ÓCULOS (gafas), PICOLÉ (paleta), CHOCOLATE (chocolate), CAMINHÃO (camión), GARRAFA (botella), AZUL (azul).	2,5
GUARDA-ROUPA (armário), PORCO (cerdo), SUJO (sucio), GUARDA-CHUVA (paraguas), COBERTOR (cobija), AMARELO (amarillo), MORANGO (fresa), PIA (lavamanos), COMPUTADOR (computadora), UMBIGO (ombligo), BANHEIRA (bañera), AREIA (arena), BERÇO (cuna), COELHO (conejo).	2,6
BORBOLETA (mariposa), PIJAMA (pijama), UVA (uva), PAPEL (papel), LÁPIS (lápiz), CHUVEIRO (ducha), IRMÃO/IRMÃ (hermano/hermana), BLUSA (blusa), SALA (sala), FOTO (foto), VASSOURA (escoba), VESTIR (vestir), PIPOCA (palomitas), CHINELO (sandalia), BOLSA (bolso), CARNE (carne), NUVEM (nube), MACACO (mono), CASACO (abrigo).	2,7
AVIÃO (avión), ESCOVA (cepillo), CAMISETA (camiseta), LARANJA (naranja), LEÃO (león), GELADEIRA (refrigerador), CHAPÉU (sombrero), ORELHA (oreja), CHAVE (llave), SABONETE (jabón), QUARTO (cuarto), SAPO (sapo), LÍNGUA (lengua).	2,8
ÁRVORE (árbol), JANELA (ventana), PEDRA (piedra), TELEFONE (teléfono), MENINA/MENINO (niña/niño), MAÇA (manzana), FOGÃO (estufa), PASSARINHO (pajarito), BICICLETA (bicicleta), BOLO (pastel), TOALHA (toalla), PEIXE (pez), COMIDA (comida), PENTEAR (peinar), ESCORREGADOR (resbaladero).	2,9
CALÇA (pantalón), COZINHA (cocina), PATO (pato), BALANÇO (columpio), LIVRO (libro), SAPATO (zapato), LEITE (leche), DOENTE/DODÓI (enfermo/dolorido), ESTRELA (estrella), CADEIRA (silla), PORTA (puerta), TRAVESSEIRO (almohada), VACA (vaca), MESA (mesa), ESCOVA DE DENTE (cepillo de dientes), CAVALO (caballo), LUA (luna), FACA (cuchillo).	3,0
GALINHA (gallina), DENTE (diente), PÃO (pan), SOFÁ (sofá), FEIJÃO (frijol), BANHEIRO (baño), GARFO (tenedor), OVO (huevo), BOLACHA/BISCOITO (galleta), BALÃO (globo), ROUPA (ropa), PRATO (plato), COMIDA (comida), MEIA (media), NENÉM (bebé).	3,1
PERNA (pierna), FLOR (flor), TELEVISÃO (TV) (televisión), AVÔ/AVÓ (abuelo/abuela), BUMBUM (trasero), DEDO (dedo), BICO, CHUPETA (chupete), COCÔ (caca), SUCO (jugo), COLHER (cuchara), DORMIR (dormir).	3,2
ARROZ (arroz), SOL (sol), CAMA (cama), OLHO (ojo), FRALDA (pañal), BONECA (muñeca), CABELO (cabello), BANANA (banana), COPO (vaso), COMER (comer), BRAÇO (brazo), MÃO (mano), NARIZ (nariz), MAMADEIRA (biberón), GATO (gato), CABEÇA (cabeza).	3,3
CARRO (carro), PAI/PAPAI (papá), CACHORRO (perro), CASA (casa), BARRIGA (barriga), PÉ (pie)	3,4
BOLA (pelota), BOCA (boca), ÁGUA (agua), MÃE/MAMÃE (mamá).	3,5

**Tabla 2:** Descripción de las palabras y valor medio del análisis de representatividad

**Palabras/ PALAVRAS**	**M**
AREIA (arena), NUVEM (nube), ROUPA (ropa), SALA (sala), FOTO (foto), VESTIR (vestir), CARNE (carne), QUARTO (cuarto), JANELA (ventana), PEDRA (piedra), COMIDA (comida), ARROZ (arroz), ÁGUA (agua), SOPA (sopa), LER (leer), SUJO (sucio), IRMÃO/IRMÃ (hermano/hermana), COMIDA (comida), PENTEAR (peinar), cocina / COZINHA, baño / BANHEIRO, COCÔ (caca), COMER (comer).	0,8
PENTE (peine), PROFESSORA (profesora), PISCINA (piscina),ELEFANTE (elefante), PICOLÉ (paleta), AZUL (azul), GUARDA-ROUPA (armario), COBERTOR (cobija), AMARELO (amarillo), PIA (lavamanos), BERÇO (cuna), BORBOLETA (mariposa) PIJAMA (pijama), PAPEL ( papel), LÁPIS (lápiz), CHUVEIRO (ducha), BLUSA (blusa), PIPOCA (palomitas), CHINELO (sandalia), CASACO (abrigo), CAMISETA (camiseta), CHAVE (llave), SABONETE (jabón), LÍNGUA (lengua), TOALHA (toalla), ESCORREGADOR (resbaladero), CALÇA (pantalón), BALANÇO (columpio), ESTRELA (estrella), CADEIRA (silla), TRAVESSEIRO (almohada), ESCOVA DE DENTE (cepillo de dientes), LUA (luna), FLOR (flor), DORMIR (dormir), SOL (sol), FRALDA (pañal), AVÔ/AVÓ (abuelo/abuela), PAI/PAPAI (papá), MÃE/MAMÃE (mamá) .	0,9
BATATA (papa), XÍCARA (taza), MOTO (moto),ÓCULOS (gafas), CHOCOLATE (chocolate), CAMINHÃO (camión), GARRAFA (botella), PORCO (cerdo), GUARDA-CHUVA (paraguas), MORANGO (fresa), COMPUTADOR (computadora), UMBIGO (ombligo), BANHEIRA (bañera), COELHO (conejo), UVA (uva), VASSOURA (escoba), BOLSA (bolso), MACACO (mono), AVIÃO (avión), ESCOVA (cepillo), LARANJA (naranja), LEÃO (león), GELADEIRA (refrigerador), CHAPÉU (sombrero), ORELHA (oreja), SAPO (sapo), ÁRVORE (árbol), TELEFONE (teléfono), MENINA/MENINO (niña/niño), MAÇA (manzana), FOGÃO (estufa), PASSARINHO (pajarito), BICICLETA (bicicleta), BOLO (pastel), PEIXE (pez), PATO (pato), LIVRO (libro), SAPATO (zapato), LEITE (leche), DOENTE/DODÓI (enfermo/dolorido), PORTA (puerta), VACA (vaca), MESA (mesa), CAVALO (caballo), FACA (cuchillo), GALINHA (gallina), DENTE (diente), PÃO (pan), SOFÁ (sofá), FEIJÃO (frijol), GARFO (tenedor), OVO (huevo), BOLACHA/BISCOITO (galleta), BALÃO (globo), PRATO (plato), MEIA (media) NENÉM (bebé), PERNA (pierna), TELEVISÃO (TV) (televisión), BUMBUM (trasero), DEDO (dedo), BICO/CHUPETA (chupete), SUCO ( jugo), COLHER (cuchara), CAMA (cama), OLHO (ojo), BONECA (muñeca), CABELO (cabello), BANANA (banana), COPO (vaso), BRAÇO (brazo), MÃO (mano), NARIZ (nariz), MAMADEIRA (biberón), GATO (gato), CABEÇA (cabeza), CARRO (carro), CACHORRO (perro), CASA (casa), BARRIGA (barriga), PÉ (pie), BOLA (pelota), BOCA (boca).	1,0

### Análisis de los datos

De acuerdo con las respuestas de los jueces para generar la lista de palabras que conformará el banco de palabras, se realiza un análisis descriptivo a través de la distribución de la media de medias de las 480 palabras, teniendo en cuenta el valor de la media de medias en cuanto a familiaridad y representatividad. Las palabras que no estuvieran en este rango fueron descartadas.

El análisis de los datos producto del resultado de esta investigación, fue de tipo cuantitativo, descriptivo e inferencial. Los datos fueron analizados mediante el software R (*R Core Team)*, específicamente con la ayuda de paquetes diseñados para la minería de datos textuales (SnowballC, tmandwordcloud2). Las palabras presentadas por los tres grupos de jueces se presentaron en tablas de frecuencia y también mediante la técnica de ilustración de nubes de palabras generada por el software. Las palabras familiares y representativas más utilizadas se resaltan en la nube de acuerdo con posición, color y tamaño.

Quedando organizadas de la siguiente forma:

● El ***tamaño*** de la palabra está dado por la ***representatividad*.** Es decir que, cuanto más grande es la palabra en la nube, es considerada más representativa. Las palabras grandes indican conceptos centrales, típicos o definidores del contexto. Las palabras pequeñas son menos representativas tal vez más periféricas o neutrales.

● El ***color*** de la palabra representa la ***familiaridad*** . Este varia en un grado continuo (de azul oscuro a amarillo/blanco). Entre más claro este el color(ejemplo, amarillo verdoso) indica más familiaridad. Los colores más oscuros (ejemplo, morado/azul oscuro) indican baja familiaridad.

## RESULTADOS

Los grupos de jueces que participaron en la consolidación de este banco de palabras se dividieron de acuerdo con la cantidad de respuestas emitidas, ya que no todos los jueces respondieron a las dos preguntas, es por esto por lo que quedaron conformados así: 21 jueces para familiaridad, los cuales estaban conformados por 9 fonoaudiólogos expertos en el área, 6 fonoaudiólogos madres de niños y 6 madres no fonoaudiólogas. En la representatividad fueron 13 en total, los cuales estaban conformados por 7 fonoaudiólogos expertos en el área, 3 fonoaudiólogas madres de niños y 3 madres no fonoaudiólogos.

De acuerdo con los criterios de selección de las palabras que conformaron el nuevo banco de palabras, se seleccionaron solo aquellas que cumplieran con los valores dados a partir de la media de las medias. Es decir, que, de acuerdo con la respuesta de los jueces, solo se tomaron las palabras que al ser familiares estaban dadas por un valor de media mayor a 2,5 y para ser representativas con un valor de media mayor a 0,2.

Los resultados de la Figura 1 representan el criterio de selección de las palabras cuyo valor de media estuviera por encima de 2, 5. Mientras que la Figura 2 representa el criterio de selección de las palabras cuyo valor de media estuviera por encima de 0,2.

**Figura 1:** Curva de distribución de las medias en cuanto a familiaridad

**Figura 2:** Curva de distribución de las medias en cuanto a representatividad

Las palabras se muestran en portugués brasileño, dado que la recolección, evaluación y análisis se realizaron con población infantil hablante de este idioma, preservando la validez cultural y lingüística del material.

El total de palabras seleccionadas a partir de la respuesta de los jueces fue de 146 de acuerdo con los valores de media tanto de familiaridad como de representatividad. En las tablas se distribuyen de acuerdo con la familiaridad y representatividad.

Las palabras familiares y representativas fueron distribuidas como se observa:

En la Tabla 1 muestra la lista de palabras y valor medio desde la familiaridad. Mientras que la Tabla 2 lista las palabras y el valor medio desde la representatividad. En la Figura 3, las palabras están representadas por colores y tamaños, siendo tamaño grande las más representativas y de color más claro las más familiares.

**Figura 3:** Distribución de las palabras familiares y representativas

Ademas se identifican, en primer lugar, palabras de alta representatividad y familiaridad, reconocibles por su gran tamaño y colores claros, cercanos al amarillo. Dentro de esta categoría, y según su pertenencia semántica, se incluyen en partes del cuerpo: *pé* (pie), *mão* (mano), *boca* (boca) y *olho* (ojo); en animales: *gato* (gato) y *pato* (pato); en objetos y utensilios: *copo* (vaso), *pente* (peine) y *bola* (pelota); en alimentos: *ovo* (huevo) y *suco* (jugo); y en elementos del entorno: *sol* (sol) y *casa* (casa). Estas palabras ocupan posiciones centrales en la nube y presentan alta visibilidad.

En segundo lugar, se observan términos de alta representatividad, pero menor familiaridad, caracterizados por gran tamaño y colores más oscuros, con matices violetas o azulados. Entre ellos se encuentran, en alimentos: *uva* (uva) y *maçã* (manzana); en animales: *leão* (león); en objetos: *livro* (libro) y *faca* (cuchillo); y en acciones o conceptos: *ler* (leer). Aunque mantienen una posición destacada en la disposición general, su color indica menor grado de familiaridad reportado por los jueces.

En tercer lugar, aparecen palabras de menor representatividad, pero alta familiaridad, identificadas por su tamaño reducido y tonalidad clara. Aquí se incluyen, en alimentos: *banana* (banana), *bolo* (pastel) y *arroz* (arroz); en animales: *cachorro* (perro) y *galinha* (gallina); en objetos: *cama* (cama) y *prato* (plato). Estos términos se distribuyen mayoritariamente en la periferia de la nube, manteniendo, no obstante, una coloración indicativa de alta familiaridad.

Por último, se distinguen palabras de baja representatividad y familiaridad, que combinan tamaño pequeño y colores oscuros. Entre ellas se encuentran, en objetos y utensilios: *escorregador* (resbaladera), *travesseiro* (almohada) y *guarda-chuva* (paraguas); en acciones: *pentear* (peinar) y *vestir* (vestir); y en conceptos o expresiones infantiles: *dodói* (dolor) y *bumbum* (trasero). Su presencia es menor y su ubicación en la periferia refleja la escasa prominencia visual en la nube.

## DISCUSIÓN

El proceso de validación mediante jueces tanto expertos como cuidadores, en este caso madres con o sin conocimiento en el área de fonoaudiología, es fundamental para garantizar que las palabras seleccionadas sean relevantes tanto desde una perspectiva técnica como contextual.

Al combinar las percepciones de fonoaudiólogos con experiencia, las de madres con trayectoria en el área de fonoaudiología y madres simplemente, genera una validez de contenido sólido que refleja el uso real del lenguaje infantil.

Esta práctica es recomendada en validaciones transculturales de instrumentos de evaluación, como se evidencia en estudios como PEEPS‑BP, donde se emplearon métodos similares con éxito para asegurar representatividad y fidelidad semántica en el contexto brasileño^(7)^_._

Estudios han demostrado que las características de comunicación dirigida por el niño (*Comunicação Dirigida para a Criança - CDC*) apoyan la adquisición del lenguaje tanto en la comprensión como en la producción. Por lo tanto, estas nuevas normas de asociación de palabras orientadas a niños proporcionan una perspectiva novedosa sobre el contexto semántico de los niños pequeños y el desarrollo léxico temprano^(12)^.

La inclusión de criterios como seleccionar palabras con familiaridad y representatividad optimiza la calidad del banco de palabras para un niño. La familiaridad léxica ha demostrado ser un predictor robusto del desarrollo del lenguaje temprano incluyendo vocabulario expresivo y fonológico en edades tempranas^(13)^.

Incorporar madres permite captar aspectos culturales y semánticos no siempre evidentes en literatura técnica. Investigaciones en intervención temprana han mostrado que la convergencia entre visión profesional y perspectiva maternal mejora la adhesión familiar y la eficacia del tratamiento. Estudios sobre desarrollo léxico de primera infancia revelan que la familiaridad con las palabras predice mejor la adquisición que variables como frecuencia o longitud de palabra^(14)^.

A su vez, la focalización en palabras con alta familiaridad y baja representatividad garantiza que el banco sea accesible, pero discriminativo para el grupo etario. Por ello la relación a los resultados de las palabras, que normalmente van asociadas a los elementos y lugares del hogar.

En Brasil, existen varios inventarios validados que miden repertorios léxicos infantiles, como los estudios sobre vocabulario a los 24 y 30 meses basados en el Inventario de MacArthur y otros desarrollados localmente^(4,6-8)^. Sin embargo, la mayoría carecen de una selección de ítems basada en criterios de familiaridad y representatividad validados por jueces expertos y madres con experiencia en el área.

Si bien los criterios aplicados delimitaron 146 palabras, la inclusión de palabras con familiaridad y representatividad fuera de umbrales podría aumentar el rango de evaluación. La creación de este banco de palabras, basada en juicios expertos y criterios cuantitativos sólidos, representa una herramienta práctica y fundamentada científicamente^(15)^_._

No solo promueve una evaluación adaptada para la población brasileña, sino que también fomenta mejores prácticas clínicas en fonoaudiología temprana. De esta forma, las 146 palabras resultantes se alinean con los márgenes de edad adecuados y aseguran que cada término sea conocido y útil para la evaluación clínica. El instrumento diseñado permite a los fonoaudiólogos evaluar de forma rápida y específica el repertorio léxico de niños pequeños.

La selección de vocabulario debe anclarse en un enfoque bioecológico que incorpore la edad cronológica, el contexto cultural y el entorno familiar del niño. Bronfenbrenner postula que la interacción dinámica entre factores internos y ambientales moldea el desarrollo lingüístico^(16)^_._

Estudios contemporáneos en niños sudafricanos y canadienses confirman que el tipo de input lingüístico en ocasiones medido mediante interacción verbal en el hogar, exposición bilingüe o prácticas de lectura compartida es un predictor robusto de la amplitud y profundidad del vocabulario receptivo y expresivo^(17,18)^_._

Estudios recientes demuestran que tanto la frecuencia como la precisión de una palabra incrementan su probabilidad de ser comprendida y producida por niños entre 16 y 30 meses, con interacciones sinérgicas que decaen con la edad^(19)^. Un metaanálisis sobre la edad en la adquisición indica que términos adquiridos a temprana edad, se procesan con mayor rapidez y precisión^(20)^.

Este nuevo banco de 146 palabras completa esa brecha (Apéndice A), ofreciendo un instrumento con evidencia de validez de contenido, aplicado en etapas clínicas y respaldado por análisis estadísticos. Ello enfatiza que las palabras incluidas en evaluaciones deben reflejar tanto el conocimiento pasivo como la capacidad de uso activo en contextos comunicativos reales, promoviendo la relación funcional del lenguaje entre ámbitos familiares, escolares y culturales^(21)^_._

Estudios recientes indican que herramientas fonológicas adaptadas al contexto lingüístico y cultural de Brasil favorecen resultados más precisos y eficaces en intervención fonoaudiológica. Además, disponer de un listado confiable y validado contribuye a la consistencia institucional y profesional, fomentando la evidencia en la práctica clínica.

## CONCLUSIÓN

Este trabajo de investigación permite brindar una herramienta para uso clínico de profesionales en salud. Se encontraron 146 palabras disponibles que se consideraron familiares y representativas. Este banco no solo es útil para evaluar el lenguaje temprano, sino que tiene un potencial terapéutico amplio. Las palabras seleccionadas pueden servir como estímulos para estrategias de evaluación e intervención fonológica, narrativa y comunicación aumentativa y alternativa (CAA).

## Apéndice A Banco de Palabras del Desarrollo Lingüístico Infantil (BPDLI): un Instrumento Validado para el Portugués Brasileño

Preámbulo

Este instrumento es una herramienta práctica, funcional y culturalmente validada, diseñada específicamente para profesionales de la Fonoaudiología que trabajan con la población brasileña. El **BPDLI** surge como resultado de un riguroso proceso de investigación que incluyó la recopilación y validación de un conjunto de palabras altamente representativas y familiares para niños de habla portuguesa brasileña en edades tempranas. Las palabras incluidas en este banco han sido organizadas en categorías, siguiendo la estructura del reconocido Inventario de Desarrollo Comunicativo MacArthur, adaptado a las particularidades lingüísticas y culturales de Brasil. (Ver Tabla 3).

**Tabla 3:** Banco de Palabras do Desarrollo Lingüístico Infantil (BPDLI): Instrumento Validado para el Portugués Brasileño

**PALAVRA**	**FAMILIARIDADE**	**REPRESENTATIVIDADE**	**CATEGORÍA**
COMER	3,292	0,845	**PALAVRAS DE AÇÃO**
DORMIR	3,236	0,917	
LER	2,514	0,845	
PENTEAR	2,931	0,845	
VESTIR	2,694	0,833	
BORBOLETA	2,653	0,917	**ANIMAIS**
CACHORRO	3,389	1,000	
CAVALO	3,042	1,000	
COELHO	2,639	1,000	
ELEFANTE	2,528	0,917	
GALINHA	3,056	1,000	
GATO	3,319	1,000	
LEÃO	2,778	1,000	
MACACO	2,736	1,000	
PASSARINHO	2,903	1,000	
PATO	2,972	1,000	
PEIXE	2,917	1,000	
PORCO	2,569	1,000	
SAPO	2,847	1,000	
VACA	3,014	1,000	
ÁGUA	3,500	0,833	**COMIDAS E BEBIDAS**
ARROZ	3,250	0,833	
BANANA	3,292	1,000	
BATATA	2,500	1,000	
BOLACHA/BISCOITO	3,083	1,000	
BOLO	2,903	1,000	
CARNE	2,722	0,833	
CHOCOLATE	2,528	1,000	
COMIDA	2,917	0,845	
FEIJÃO	3,069	1,000	
LEITE	2,986	1,000	
MAÇA	2,889	1,000	
MORANGO	2,597	1,000	
OVO	3,083	1,000	
PÃO	3,056	1,000	
PICOLÉ	2,528	0,917	
PIPOCA	2,708	0,917	
SOPA	2,514	0,845	
SUCO	3,222	1,000	
UVA	2,653	1,000	
AMARELO	2,583	0,917	**QUALIDADES E ATRIBUTOS**
AZUL	2,542	0,917	
DOENTE/DODÓI	2,986	1,000	
LARANJA	2,764	1,000	
SUJO	2,569	0,845	
BALÃO	3,097	1,000	**BRINQUEDOS**
BOLA	3,500	1,000	
BONECA	3,292	1,000	
LÁPIS	2,667	0,917	
LIVRO	2,986	1,000	
CASA	3,403	1,000	**LUGARES FORA DE CASA**
PISCINA	2,514	0,917	
BANHEIRA	2,611	1,000	**MÓVEIS E APOSENTOS**
BANHEIRO	3,069	0,845	
BERÇO	2,625	0,917	
CADEIRA	3,000	0,917	
CHUVEIRO	2,667	0,917	
COZINHA	2,958	0,845	
FOGÃO	2,889	1,000	
GELADEIRA	2,778	1,000	
GUARDA-ROUPA	2,556	0,917	
JANELA	2,861	0,833	
MESA	3,028	1,000	
PIA	2,597	0,917	
PORTA	3,000	1,000	
QUARTO	2,833	0,833	
SALA	2,694	0,833	
SOFÁ	3,056	1,000	
TELEVISÃO (TV)	3,153	1,000	
AREIA	2,625	0,774	**OBJETOS EXTERNOS**
ÁRVORE	2,861	1,000	
BALANÇO	2,972	0,917	
ESCORREGADOR	2,944	0,917	
ESTRELA	3,000	0,917	
FLOR	3,153	0,917	
LUA	3,042	0,917	
NUVEM	2,722	0,774	
PEDRA	2,875	0,833	
SOL	3,250	0,917	
BARRIGA	3,444	1,000	**PARTES DO CORPO**
BICO/CHUPETA	3,208	1,000	
BOCA	3,500	1,000	
BRAÇO	3,306	1,000	
BUMBUM	3,167	1,000	
CABEÇA	3,333	1,000	
CABELO	3,292	1,000	
COCÔ	3,222	0,845	
DEDO	3,194	1,000	
DENTE	3,056	1,000	
LÍNGUA	2,847	0,917	
MÃO	3,306	1,000	
NARIZ	3,306	1,000	
OLHO	3,264	1,000	
ORELHA	2,792	1,000	
PÉ	3,444	1,000	
PERNA	3,153	1,000	
UMBIGO	2,611	1,000	
AVÔ/AVÓ	3,167	0,929	**PESSOAS**
IRMÃO/IRMÃ	2,667	0,845	
MÃE/MAMÃE	3,500	0,929	
MENINA/MENINO	2,875	1,000	
NENÉM	3,139	1,000	
PAI/PAPAI	3,375	0,929	
PROFESSORA	2,500	0,917	
BLUSA	2,694	0,917	**ROUPAS E ACCESORIOS**
BOLSA	2,708	1,000	
CALÇA	2,958	0,917	
CAMISETA	2,764	0,917	
CASACO	2,736	0,917	
CHAPÉU	2,792	1,000	
CHINELO	2,708	0,917	
FRALDA	3,278	0,917	
GUARDA-CHUVA	2,583	1,000	
MEIA	3,125	1,000	
ÓCULOS	2,528	1,000	
PIJAMA	2,653	0,917	
ROUPA	3,097	0,774	
SAPATO	2,986	1,000	
COMIDA	3,097	0,833	**ROTINA DIARIA**
CAMA	3,250	1,000	**UTENSILIOS DE CASA**
CHAVE	2,806	0,917	
COBERTOR	2,583	0,917	
COLHER	3,236	1,000	
COMPUTADOR	2,597	1,000	
COPO	3,292	1,000	
ESCOVA	2,750	1,000	
ESCOVA DE DENTE	3,028	0,917	
FACA	3,042	1,000	
FOTO	2,694	0,833	
GARFO	3,069	1,000	
GARRAFA	2,542	1,000	
MAMADEIRA	3,306	1,000	
PAPEL	2,653	0,917	
PENTE	2,500	0,917	
PRATO	3,097	1,000	
SABONETE	2,806	0,917	
TELEFONE	2,875	1,000	
TOALHA	2,903	0,917	
TRAVESSEIRO	3,000	0,917	
VASSOURA	2,694	1,000	
XÍCARA	2,500	1,000	
AVIÃO	2,750	1,000	**VEICULOS**
BICICLETA	2,903	1,000	
CAMINHÃO	2,542	1,000	
CARRO	3,361	1,000	
MOTO	2,514	1,000	

Objetivo

El principal objetivo del **BPDLI** es ofrecer a los fonoaudiólogos una base sólida, tanto clínica como funcional, para optimizar la planificación de intervenciones, monitorear el progreso del desarrollo del lenguaje y del habla, y seleccionar vocabulario pertinente durante el proceso terapéutico.

Instrucciones de Uso

El **BPDLI** ha sido concebido como una guía de referencia fundamental para la evaluación del lenguaje expresivo en niños de primera infancia. Para su aplicación, el profesional fonoaudiólogo podrá utilizar la lista de palabras para identificar aquellas que el niño reconoce como familiares y las que son representativas de su repertorio lingüístico activo. Esta información es crucial para establecer objetivos terapéuticos individualizados y diseñar estrategias de intervención ajustadas a las necesidades específicas de cada niño.
